# Residual Jaw Bone Volume and Five-Year Outcomes of Custom-Made Subperiosteal Implants: A Retrospective Study

**DOI:** 10.3390/jfb17100482

**Published:** 2026-09-22

**Authors:** Jan Łoginoff, Zuzanna Popińska, Agata Majos, Marcin Elgalal

**Affiliations:** 1 II Department of Radiology and Diagnostic Imaging, Central Clinical Hospital, Medical University of Lodz, 251 Pomorska Street, 92-213 Lodz, Poland; jan.loginoff@office365.umed.pl (J.Ł.);; 2 Department of Maxillofacial Surgery, Central Clinical Hospital, Medical University of Lodz, 251 Pomorska Street, 92-213 Lodz, Poland

**Keywords:** subperiosteal implant, dental implants, alveolar bone loss, jaw, edentulous, cone-beam computed tomography, printing, three-dimensional, titanium, retrospective studies, treatment outcome

## Abstract

Background: Custom subperiosteal implants represent an alternative for severe jaw atrophy, yet long-term quantitative bone predictors remain limited. This retrospective study aimed to evaluate 5-year implant survival and determine preoperative volumetric bone thresholds using 3D CT segmentation. Methods: The unit of analysis was individual implant sites in 40 patients (25 maxillae and 15 mandibles). Primary outcomes were 5-year cumulative implant survival (Kaplan–Meier method) and preoperative total jaw bone volume (cm^3^) measured via standardized 3D segmentation using BrainLab software (version 1.7.0.116). Group comparisons between surviving and failed sites were performed with 95% confidence intervals (CIs). Results: Overall 5-year Kaplan–Meier implant survival was 77.5% (31/40; 95% CI: 64.6–90.4%). Maxillary sites achieved a survival rate of 80.0% (20/25; 95% CI: 64.3–95.7%), whereas mandibular sites achieved 73.3% (11/15; 95% CI: 51.0–95.6%). Preoperative jaw bone volume was significantly lower in failure cases across both maxillary and mandibular cohorts (*p* < 0.05). Nine implants failed due to biological (*n* = 4), mechanical (*n* = 2), or combined (*n* = 3) complications. Conclusions: Residual bone volume exhibits a statistically significant association with framework survival, whereas the categorical Cawood and Howell classification showed no statistically significant association in this cohort. These findings suggest that continuous volumetric assessment may serve as a useful exploratory parameter for surgical risk stratification, though predictive superiority requires validation in larger, adjusted multivariable models.

## 1. Introduction

The rehabilitation of patients with severe maxillary or mandibular atrophy remains one of the most significant challenges in implant dentistry. In cases of advanced residual ridge resorption (e.g., Cawood and Howell Class IV–VI), conventional endosseous implants are often unfeasible due to insufficient bone volume and unfavorable prosthetic relationships [1,2]. While reconstructive procedures like autologous grafting or sinus lifts are effective, they are frequently rejected by elderly or medically compromised patients due to high morbidity, cost, and extended treatment times. Subperiosteal implants serve as a critical graftless alternative in these complex scenarios [3]. Unlike endosseous systems, these custom-made titanium frameworks are placed directly beneath the periosteum, resting on the surface of the residual bone and secured with osteosynthesis screws. Historically, this concept faced criticism due to poor fit and unpredictable outcomes [4]; however, the integration of CBCT imaging, CAD/CAM technology, and additive manufacturing has revitalized the field [5,6,7,8]. Modern subperiosteal implants are now digitally designed to match the patient’s unique anatomy with high precision. This allows for optimized load distribution and strategic fixation [5,6,7,8].

The primary objective of this study was to analyze the risk factors affecting the survival rate of custom-made subperiosteal implants, with a specific focus on volumetric bone assessment. In current clinical practice, the Cawood and Howell classification remains the most widely used tool for evaluating jaw atrophy [1]. However, its application can be subjective, and the nuances between different classes are often prone to misinterpretation during radiological analysis. Furthermore, this qualitative scale does not account for the significant anatomical variations found within the same category. To address these limitations, we propose volumetric analysis as an objective, precise, and reproducible method for quantifying residual bone volume. By shifting from descriptive classifications to measurable data, we aim to determine whether specific volumetric thresholds can serve as reliable predictors for implant success or failure.

## 2. Methods

### 2.1. Clinical Data

#### 2.1.1. Patient Selection and Cohort Flow

Patient selection and study flow were structured in accordance with the Strengthening the Reporting of Observational Studies in Epidemiology (STROBE) guidelines (Figure 1). A total of 86 patients treated with custom-made subperiosteal titanium implants between 2015 and 2020 were retrospectively screened. To ensure high methodological rigor and eliminate within-patient clustering, strict inclusion and exclusion criteria were applied a priori. The inclusion criteria were: (1) adult patients (age > 18 years) presenting with severe maxillary or mandibular atrophy (Cawood and Howell Class IV–VI); (2) treatment with a single custom-made DMLS titanium subperiosteal implant; (3) availability of complete preoperative high-resolution CBCT/CT datasets in DICOM format suitable for 3D volumetric bone assessment; and (4) a fully documented, uninterrupted 5-year post-surgical follow-up period. Out of the 86 screened patients, 46 were excluded for the following specific reasons: incomplete preoperative radiological documentation or incompatible CT parameters (*n* = 14) and bilateral subperiosteal implant placement (*n* = 8, excluded to avoid intra-individual data clustering and outcome bias). To ensure complete 5-year clinical and radiological data, a complete-case analysis approach was utilized. Patients lost to follow-up or relocated prior to the 5-year endpoint (*n* = 12) were excluded from the primary analytical cohort (N = 40). Primary survival estimates thus represent the 5-year complete-case cumulative proportion. Patients with uncontrolled systemic conditions severely impairing bone metabolism or soft-tissue healing (*n* = 7) and a history of previous extensive jaw reconstructive procedures with free vascularized bone flaps (*n* = 5) were also excluded. Consequently, the final study cohort comprised 40 individual patients (26 men, 14 women; median age 63.5 years), with each patient contributing exactly one treatment site (25 maxillae and 15 mandibles) as the primary unit of analysis. The index date was defined as the day of surgical placement and fixation of the subperiosteal framework.

#### 2.1.2. Clinical Endpoints and Operational Definitions

Clinical outcomes were adjudicated independently by two senior maxillofacial surgeons using standardized, rigorous criteria. Endpoints were categorized as follows. Implant Success: Complete stability of the subperiosteal framework, absence of persistent pain or discomfort, complete mucosal coverage without dehiscence or framework exposure, absence of peri-implant infection, and stable osteosynthesis screw positioning without progressive peri-screw bone resorption. Implant Survival: Maintenance of the framework and functional prosthetic load at 5 years, including cases that experienced minor transient complications (e.g., localized soft-tissue dehiscence or single screw loosening) successfully managed via minor surgical reintervention (mucosal flap revision or screw removal) without requiring full framework explantation. Implant Failure: Unrecoverable biological or mechanical failure requiring total surgical removal/explantation of the subperiosteal framework. Specific complication types were recorded, including soft-tissue dehiscence/framework exposure, transient mucosal inflammation, screw loosening, and structural framework fracture.

### 2.2. Radiological Protocol and Volumetric Segmentation

Preoperative radiological evaluation was performed using high-resolution cone-beam computed tomography (CBCT) datasets acquired on a single dedicated scanner (NewTom VGi evo, Cefla, Imola, Italy). Scans were acquired using a standardized protocol with a large field of view (FOV, 16 × 13 cm) at 110 kVp, 3–15 mA (pulsed mode), and an isotropic voxel size of 0.3 mm. Three-dimensional volumetric bone segmentation and quantitative analysis were executed using BrainLab software (version 1.7.0.116; Brainlab AG, Munich, Germany). Anatomical boundaries for regions of interest (ROIs) were defined as follows: Maxillary ROI: Bounded superiorly by the hard palate and nasal floor, laterally and anteriorly by the outer cortical surfaces of the alveolar process and zygomaticomaxillary buttresses, and posteriorly by the pterygomaxillary junction. The maxillary sinus cavities, nasal airway, and incisive canal were explicitly excluded from the skeletal volume calculation. Mandibular ROI: Bounded by the superior alveolar crest, inferior basal border, posterior ramus limits, and outer buccal/lingual cortical plates. The mandibular canal, mental foramina, and soft-tissue spaces were excluded. Tooth/Artifact Protocol: Dental crowns, high-density metallic restorations, and streaking artifacts were manually isolated and excluded from bone thresholds using slice-by-slice manual editing across coronal and axial planes. Initial bone segmentation was generated using intensity thresholding calibrated for cortical bone (Hounsfield units/gray values adjusted per scan), followed by systematic manual correction across all coronal and axial slices by two independent board-certified radiologists. To assess inter-observer reliability, both radiologists independently performed volumetric measurements on all included examinations (N = 40). Reliability was evaluated using a two-way random-effects model, absolute agreement, average-measures intra-class correlation coefficient [ICC(2, k)]. Inter-observer reliability demonstrated strong consistency, with an ICC of 0.89 (95% CI: 0.82–0.94) for maxillary volume and 0.87 (95% CI: 0.79–0.93) for mandibular volume. The mean of the two independent measurements was utilized for statistical analyses to reduce random measurement error. A representative example of the segmentation output is shown in Figure 2.

### 2.3. Biomaterial Specifications and Standardized Treatment Protocol

#### 2.3.1. Treatment Protocol

Patient-specific subperiosteal devices evaluated in this study were fabricated between 2015 and 2020 by two certified medical implant manufacturers: ChM sp. z o.o. (Juchnowiec Koscielny, Poland; *n* = 26/40, 65.0%) and Medgal sp. z o.o. (Bialystok, Poland; *n* = 14/40, 35.0%). Patient-specific 3D CAD models were designed from high-resolution DICOM CT/CBCT datasets (slice thickness ≤ 0.625 mm). Initial 3D bone segmentation was performed using dedicated medical imaging software (3D Slicer version. 5.6.2/BrainLab for anatomical landmarking and thresholding), and then CAD modeling was executed using specialized implant design software (Geomagic Freeform version 2014, 3D Systems, Rock Hill, SC, USA). Frameworks were additively manufactured from medical-grade Titanium-6Aluminum-4Vanadium Extra Low Interstitial alloy powder (Ti-6Al-4V ELI, Grade 23; ISO 5832-3 [9]/ASTM F136 [10] compliant). Manufacturing was executed via Laser Powder Bed Fusion (LPBF/DMLS) using industrial systems: EOSINT M 280 (EOS GmbH, Krailling, Germany; utilized for *n* = 26 cases) and Concept Laser M2 (Concept Laser GmbH, Lichtenfels, Germany; utilized for *n* = 14 cases). Process parameters were set according to validated manufacturer protocols adhering to ISO/ASTM 52900 [11] standards, utilizing a layer thickness of 30 µm under a protective argon gas atmosphere (oxygen content < 0.1%).

#### 2.3.2. Post-Processing, Surface Metallurgy, and Sterilization

Following laser sintering, stress-relief thermal annealing was performed in a vacuum furnace (730 °C ± 10 °C for 120 min, argon cooling) to eliminate residual thermal stresses. Devices were detached from build platforms via wire electrical discharge machining (EDM) with manual support removal. Surface finishing for all frameworks incorporated a dual-zone surface protocol. Bone-contact surface: Micro-corundum blasted with high-purity Al_2_O_3_ particles (120 µm) at 4 bar to achieve a moderately rough surface profile suitable for secondary mechanical interlock. Trans-mucosal posts and neck regions: Mechanically polished to a smooth mirror finish (Ra < 0.35 µm) to minimize bacterial adhesion. Frameworks underwent chemical passivation (30% HNO3 for 30 min, ASTM F860 [12]), ultrasonic cleaning in enzymatic detergent and deionized water, and terminal steam sterilization (134 °C for 18 min, EN ISO 17665-1 [13]) prior to surgical implantation.

#### 2.3.3. Structural Architecture and Fixation Hardware

The framework architecture comprised primary struts (thickness: 1.3–1.5 mm, width: 2.2–2.5 mm) following dense cortical trajectories of the zygomaticomaxillary buttresses and canine fossae in the maxilla, or the basal border and mental pillars in the mandible. Secondary interconnecting struts had a thickness of 1.0–1.2 mm. Each device featured 4 to 6 integrated percutaneous prosthetic posts (height: 4.5 mm, external diameter: 3.5 mm) with internal threads for screw-retained prosthetics. Rigid primary osteosynthesis was secured using 4 to 8 self-tapping bicortical osteosynthesis screws (Grade 5 Ti-6Al-4V; diameter: 1.5 mm or 2.0 mm; length: 5.0–9.0 mm; manufactured by ChM/Medgal/Synthes, ISO 5835 [14] compliant) placed in pre-planned osteotomy sites away from anatomical risk zones.

#### 2.3.4. Surgical, Antibiotic, and Prosthetic Protocols

All surgical procedures were performed under local anesthesia by two experienced oral and maxillofacial surgeons (each with >15 years of reconstructive surgery experience). Antibiotic and Antiseptic Protocol: Oral Amoxicillin/Clavulanate (1.0 g) was given 30 min preoperatively; postoperatively, oral Amoxicillin/Clavulanate (1.0 g BID) was maintained for 7 days (or Clindamycin 600 mg TID in penicillin-allergic patients). Preoperative 0.2% Chlorhexidine digluconate mouthwash was performed for 2 min and continued BID for 14 days post-surgery. Soft-Tissue Management: Full-thickness mucoperiosteal flaps were reflected beyond the mucogingival junction to expose cortical bone. Periosteal releasing incisions were systematically performed to allow tension-free primary soft-tissue closure over the framework using 4-0/5-0 monofilament sutures (Glycolon/PTFE). Prosthetic Loading and Maintenance: Immediate non-functional prosthetic loading was implemented within 48–72 h using a CAD/CAM provisional PMMA full-arch screw-retained bridge. Definitive prosthetic loading with a CAD/CAM titanium composite or monolithic zirconia bridge occurred at 3–6 months post-op. Patients were enrolled in a mandatory professional hygiene and clinical control protocol at 1, 3, 6, and 12 months, and bi-annually thereafter for 5 years.

### 2.4. Statistics

Statistical analysis was performed using IBM SPSS Statistics (Version 27.0; IBM Corp., Armonk, NY, USA) and GraphPad Prism (Version 9.0). The primary unit of analysis was the individual subperiosteal implant site (N = 40). Two-tailed *p*-values < 0.05 were considered statistically significant for all analyses. Continuous variables were evaluated for normality using the Shapiro–Wilk test. Data are presented as medians with interquartile ranges (IQRs) due to boundary constraints and non-normal distributions in subgroups, with means and standard deviations (SDs) provided where appropriate. Categorical variables are reported as absolute counts and percentages. Bivariate comparisons of continuous variables between surviving (*n* = 31) and failed (*n* = 9) cohorts were conducted using the non-parametric Mann–Whitney U test. Categorical proportions were compared using Fisher’s exact test (for 2 × 2 tables). Cumulative 5-year implant survival was evaluated using the Kaplan–Meier product-limit method for the complete-case cohort (N = 40). The event was defined strictly as total framework explantation. Numbers at risk were reported at 12-month intervals. For subgroup comparisons of residual bone volume across Cawood and Howell classes, non-parametric effect sizes (r) were calculated using the formula $r=\frac{\left|Z\right|}{\surd N}$ where Z is the standardized test statistic from the Mann–Whitney U test and N is the total subgroup sample size. Effect sizes were interpreted according to Cohen’s criteria (0.1 = small, 0.3 = medium, 0.5 = large). All subgroup comparisons were prespecified as hypothesis-generating exploratory secondary analyses; thus, adjustment for multiplicity was deliberately omitted to preserve statistical power and prevent Type II errors in this pilot cohort. Missing data protocols were unnecessary as complete 5-year follow-up data were available for all 40 included patients (0% missingness).

### 2.5. Ethical Approval

The study was conducted in accordance with the Declaration of Helsinki. Written informed consent for surgical treatment, pre- and postoperative radiological diagnostics (CBCT/CT), and the secondary use of anonymized clinical data for research purposes was routinely obtained from all patients at the time of treatment (2015–2020). Formal ethical approval for this retrospective study protocol was granted by the Bioethics Committee of the Medical University of Łódź (Approval No. RNN/135/24/KE) in 2024. The Bioethics Committee explicitly waived the requirement to obtain additional specific informed consent for this retrospective data analysis, as all procedures were performed as part of standard clinical care and data were analyzed anonymously.

## 3. Results

### 3.1. Clinical Outcomes and Failure Etiology

The study group consisted of 40 patients, including 26 men (65%) and 14 women (35%). The median age of the participants was 63.5 years (IQR 58.00–68.00). Although the age distribution did not significantly deviate from normality (Shapiro–Wilk test, *p* = 0.119), non-parametric descriptors were reported to provide a robust summary alongside mean values.). Regarding implant localization, 25 frameworks (62.5%) were placed in the maxilla and 15 (37.5%) in the mandible. The assessment of primary stability and fit showed that the majority of patients achieved high stability scores: 22 patients (55%) scored 8 on the internal scale, 11 patients (27.5%) scored 7, and 3 patients (7.5%) scored 6. Lower stability scores were less frequent, with three patients (7.5%) scoring 5 and one patient (2.5%) scoring 4. Regarding postoperative complications and bone maintenance, 30 cases (75%) showed stable screw positioning and no significant secondary bone resorption, while 10 cases (25%) exhibited framework mobility or inflammatory signs. The overall 5-year cumulative survival rate was 77.5%, with 31 implants remaining functional in situ (Survival group) and 9 implants (22.5%) being classified as failures requiring total explantation (Failure group). Uneventful clinical success without any postoperative complications occurred in 30 cases (75.0%). One surviving case (2.5%) experienced localized soft-tissue dehiscence successfully managed via minor surgical revision, thus fulfilling the criteria for 5-year implant survival despite failing the strict definition of primary clinical success. Lifestyle and hygiene factors showed that 29 patients (72,5%) were non-smokers and 11 (27,5%) were active smokers. Oral hygiene ratings were distributed as follows: 29 patients (72.5%) received a score of 5, 5 patients (12.5%) scored 4, 4 patients (10.0%) scored 3, 1 patient (2.5%) scored 2, and 1 patient (2.5%) scored 1. Applying the prespecified cutoff (scores 4–5 vs. 1–3), 34 patients (85.0%) presented with adequate oral hygiene and 6 patients (15.0%) presented with inadequate oral hygiene.

The summary of the descriptive statistics and clinical characteristics of the study population is presented in Table 1.

All 40 included patients completed the 5-year follow-up protocol (*n* = 40, 100% complete follow-up data). Over the 60-month observation window, 31 implants remained functional, yielding an overall 5-year cumulative Kaplan–Meier survival rate of 77.5% (95% CI: 64.6–90.4%; Figure 3). A total of nine cases (22.5%) were classified as failures and underwent complete framework explantation. Uneventful healing without any clinical or radiological complications occurred in 30 cases (75%). Ten cases (25%) experienced clinical complications. In one case, minor surgical reintervention (soft-tissue flap revision for localized dehiscence) successfully salvaged the framework, allowing it to fulfill the criteria for 5-year survival. In the remaining nine cases, reinterventions failed to arrest tissue breakdown or instability, leading to framework removal. The specific causes and timing of the nine framework failures were adjudicated as follows. Biological failures (*n* = 4): Severe recurrent peri-implantitis, soft-tissue necrosis, and extensive framework exposure occurring at months 8, 14, 16, and 38 (predominantly associated with heavy smoking and poor oral hygiene). Mechanical failures (*n* = 2): Primary mechanical instability resulting from fixation screw loosening at month 22, and structural strut fracture in the canine region at month 45. Combined biological/mechanical/prosthetic failures (*n* = 3): Complex multifactorial failures involving occlusal overload with anterior strut exposure at month 11, sinus communication with maxillary sinusitis at month 18, and prosthetic abutment shear fracture at month 30. Table 2 represents the failure case data.

### 3.2. Radiological Analysis

The preoperative radiological assessment according to the Cawood and Howell classification revealed that 8 patients (20%) were categorized as Class IV, 18 patients (45%) as Class V, and 14 patients (35%) as Class VI. In terms of volumetric measurements, the median bone volume was 12.4 cm^3^ (IQR 11.82–13.3) for the maxilla and 23.45 cm^3^ (IQR 22.5–23.9) for the mandible. The volumetric assessment revealed significant associations between residual bone volume and implant survival rates. In the group of patients treated with maxillary subperiosteal implants (*n* = 25), statistically significant differences were observed in bone distribution between surviving and failed cases. For patients with surviving maxillary implants (*n* = 20; 80%), the median maxillary volume was 12.45 cm^3^ (IQR 12.13–13.25), whereas in cases of implant failure (*n* = 5; 20%), the median volume was significantly lower at 10.9 cm^3^ (IQR 10.60–11.15; Mann–Whitney U test, *p* = 0.001). In an exploratory secondary analysis, the non-index mandibular volume in this subgroup also demonstrated significant differences between surviving and failure cases (*p* = 0.001), suggesting that generalized jaw atrophy may reflect a broader systemic bone resorption phenotype. Similar trends were observed in patients who received mandibular subperiosteal implants (*n* = 15). Among surviving cases (*n* = 11; 73.3%), the median mandibular volume was 23.5 cm^3^ (IQR 22.9–24.5), compared to 21.55 cm^3^ (IQR 20.55–22.16) in the failure group (*n* = 4; 26.7%; *p* = 0.040). In exploratory evaluation, non-index maxillary bone volume in these patients was also significantly associated with mandibular implant outcomes, with a median of 13.1 cm^3^ (IQR 12.1–14.2) for success versus 11.55 cm^3^ (IQR 10.98–12.28) for failure (*p* = 0.026). These exploratory findings suggest that marked bone volume loss in both jaws may indicate a constitutional predisposition to heightened bone resorption, serving as an additional indirect indicator of implant failure risk. Figure 4 illustrates representative multi-planar visualization and baseline skeletal segmentation used for 3D bone volume quantification.

To further validate the clinical significance of bone volume, a subgroup analysis was performed comparing surviving (positive) and failed (negative) cases within the same Cawood and Howell anatomical classes. The results, summarized in Table 3, highlight significant intra-class volumetric variations that traditional qualitative assessments fail to capture.

In the maxillary group, patients categorized within the same anatomical class (Class V and Class VI) showed statistically significant differences in actual bone volume between surviving and failed outcomes. Specifically, in Class V, surviving implants were associated with a median volume of 12.7 cm^3^, while failed cases had a significantly lower volume of 11.8 cm^3^ (*p* = 0.003). A similar pattern was observed in Class VI, where failures occurred at a median volume of 10.9 cm^3^ compared to 12.0 cm^3^ in surviving cases (*p* = 0.002). Regarding the mandible, a highly significant volumetric difference was found within Class V (*p* < 0.001), where failures were associated with a reduced bone volume (median 21.9 cm^3^) compared to successful rehabilitations (median 23.7 cm^3^). In Class VI, although a lower median volume was observed for failed cases (20.7 cm^3^ vs. 22.5 cm^3^), this difference did not achieve statistical significance (*p* = 0.054). Overall, when evaluated across categorical Cawood and Howell classes, failure rates did not show statistically significant differences (*p* = 0.054). In contrast, unadjusted bivariate comparisons of baseline residual bone volume demonstrated lower volumes in the failure group (*p* = 0.018 for the maxillary cohort). These observations suggest an unadjusted association between residual volume and survival, rather than definitive predictive superiority.

## 4. Discussion

The primary finding of this study is that quantitative volumetric bone assessment using high-resolution CBCT imaging demonstrates a significant unadjusted association with subperiosteal implant survival. While the Cawood and Howell classification has traditionally served as a qualitative standard for evaluating jaw atrophy, our exploratory results indicate that categorical classification alone did not show a statistically significant relationship with framework survival in this cohort. In contrast, residual skeletal volume was significantly reduced in failure cases. However, given the bivariate and unadjusted nature of these analyses, these findings must be interpreted as hypothesis-generating observations rather than definitive evidence of predictive superiority or established clinical thresholds.

The overall 5-year cumulative survival rate of 77.5% observed in our cohort is lower than early historical series. While 77.5% of implants survived at 5 years, only 75% achieved complete, uneventful clinical success without requiring secondary soft-tissue revisions or showing signs of localized inflammation. This distinction between survival and success underscores the necessity for multi-parametric evaluation. While traditional studies often rely on qualitative descriptions, our findings highlight their inherent limitations. As shown in Table 3, successful outcomes in both the maxilla and mandible were strongly correlated with higher absolute bone volumes, even within the same anatomical class. For example, in Class V maxillary cases, failed implants occurred at a median volume of 11.8 cm^3^, whereas successful ones had 12.7 cm^3^ (*p* = 0.003). This suggests that the “high success” reported in some literature [15,16] might involve patients with higher residual volumes that qualitative scales fail to distinguish from truly borderline cases. Thus, our data supports the transition toward a quantitative volumetric threshold as a more reliable clinical predictor than the subjective Cawood and Howell classification.

Beyond volumetric measurements, our analysis identified critical lifestyle and clinical factors that significantly influence the long-term stability of subperiosteal implants. Notably, oral hygiene (*p* = 0.001) and smoking status (*p* = 0.007) emerged as the most significant predictors of failure. These findings are consistent with established literature; for instance, Moore and Hansen [5] emphasized that peri-implantitis and framework exposure are frequently exacerbated by poor plaque control and the vasoconstrictive effects of nicotine in severely atrophic ridges. Although univariate analysis revealed no statistically significant differences between the success and failure groups regarding age (*p* = 0.588) or sex (*p* = 0.694), these unadjusted baseline comparisons should be interpreted with caution. Non-significant univariate tests do not guarantee cohort homogeneity or rule out potential confounding. Given the sample size constraints and the univariable nature of these comparisons, residual confounding by unmeasured clinical or systemic variables cannot be completely excluded. The core strength of our methodology lies in the objective quantification of bone structures through advanced 3D volumetric analysis. To ensure the highest level of diagnostic precision, we utilized the BrainLab software platform, which allowed for the detailed segmentation of the residual maxillary and mandibular bone. This digital workflow is superior to traditional 2D imaging as it enables the isolation of skeletal tissues from dental artifacts and soft tissue, providing a reliable baseline for surgical planning. To address potential inter-observer variability, all volumetric measurements were performed independently by two experienced radiologists using standardized ROI boundary criteria and manual artifact exclusion. Utilizing the mean of these two independent assessments reduced random measurement error, providing a consistent quantitative baseline for statistical evaluation. By employing such a standardized and precise approach, we were able to identify subtle anatomical variations that are typically overlooked in qualitative assessments, further validating the necessity of volumetric tools in modern implantology. This methodology has already been applied in multiple different studies, confirming its viability and robustness [17,18,19].

Furthermore, it should be noted that this study intentionally focused on the volumetric bone analysis rather than the specific etiology of complications or the detailed clinical criteria for implant removal. While biological and mechanical failures led to the recorded 22.5% failure rate, the primary aim was to establish the correlation between bone volume and overall survival. From a statistical perspective, we applied relatively simple analytical methods. Given the retrospective nature of the study and the sample size of 40 cases, employing more complex multivariate models would carry a high risk of over-statistical analysis (overfitting), which could lead to misleading conclusions [20,21]. Therefore, we have exercised caution in our interpretation. It is essential to recognize that retrospective data do not allow for the establishment of definitive cause-and-effect relationships; instead, our findings should be viewed as clinical indicators. These indicators suggest a strong trend that quantitative bone volume is a decisive factor in implant stability. These results serve as a foundational pilot for future, larger-scale prospective studies that will be necessary to validate specific volumetric thresholds and refine patient selection protocols in graftless implantology.

In this study, 3D volumetric bone assessment was utilized as a preoperative prognostic predictor of subperiosteal framework survival rather than a primary clinical definition of survival. While long-term survival is evaluated via clinical criteria (e.g., framework stability, lack of infection, and functional retention), localized bone volume dictates the cortical contact area available for stress distribution and primary screw fixation. The exploratory observation that non-index, cross-arch bone volume correlated with implant survival highlights two distinct clinical factors. Biologically, pronounced volumetric loss across both jaws likely mirrors a generalized, constitutional phenotype of heightened skeletal resorption or systemic bone turnover. Biomechanically, the bone stock of the opposing arch dictates antagonist stability and occlusal force transmission; compromised opposing skeletal support can alter masticatory vector dynamics and generate unfavorable load concentrations on the subperiosteal framework. Evaluating total maxillofacial bone stock therefore provides comprehensive insight into both underlying biological susceptibility and functional occlusal biomechanics. Despite the significant findings regarding volumetric thresholds, several methodological limitations must be explicitly acknowledged. First, the retrospective, single-center design inherently limits the generalizability of our findings to broader clinical populations and varied surgical settings. Second, the exclusion of 12 patients lost to follow-up prior to the 5-year endpoint represents a complete-case analysis design, which carries an inherent risk of selection bias. Excluding lost cases rather than incorporating them as censored observations in time-at-risk models may either overestimate or underestimate overall framework survival if loss to follow-up was related to unrecorded implant complications or patient dissatisfaction. Third, the cohort selection process carries an inherent risk of selection bias. From the initial pool of 86 patients, 46 were excluded due to incomplete DICOM datasets (*n* = 14), loss to follow-up (*n* = 12), bilateral placements (*n* = 8), severe systemic conditions (*n* = 7), or prior flap reconstructions (*n* = 5). Excluding cases with missing data or incomplete follow-up may have enriched the final cohort (*n* = 40) with higher-compliance patients, potentially skewing survival estimates. Fourth, while a sample size of 40 patient sites provided adequate statistical power for univariate volumetric stratification, it remains insufficient for complex multivariable regression modeling without overfitting risks, leaving potential residual confounding by unmeasured biomechanical or systemic factors. Finally, retrospective observational data cannot establish true causality between bone volume thresholds and framework failure. The overall 5-year survival rate of 77.5% in our cohort is lower than some recent reports (which range from 92% to 100%). This difference is directly attributable to the strict operational criteria applied in our adjudication framework—where any case requiring complete explantation was classified as a failure—combined with the extreme degree of ridge atrophy (predominantly Cawood and Howell Class V and VI) in our patients. Although enforcing a strict 5-year documented follow-up requirement ensured a robust dataset free of loss-to-follow-up distortion, this retrospective selection criterion must be considered when generalizing findings. While this 5-year duration provides valuable mid-term outcome data, longer longitudinal, multi-center studies with enlarged cohorts and uniform imaging protocols are required to evaluate the ultimate lifespan of subperiosteal frameworks and externally validate preoperative volumetric cutoff values. Furthermore, while all implants adhered to identical material standards (Ti-6Al-4V ELI Grade 23) and dual-zone surface finishing protocols, manufacturing was distributed across two certified production facilities utilizing two distinct LPBF systems (EOSINT M 280 and Concept Laser M2). Although both systems operate under standardized ISO/ASTM parameters, minor inter-manufacturer variations in laser sintering dynamics or post-processing cannot be completely excluded as potential unmeasured confounders.

## 5. Conclusions

The findings of this study demonstrate an unadjusted statistical association between quantitative 3D residual bone volume and custom subperiosteal implant survival, whereas the categorical Cawood and Howell classification did not achieve statistical significance in this cohort. The observed intra-class volumetric variations suggest that continuous volumetric measurement may offer complementary descriptive detail for surgical evaluation. However, establishing whether bone volume represents an independent predictor or clinical threshold requires future prospective studies using adjusted multivariable modeling and external validation. Modifiable patient factors, particularly smoking and oral hygiene, remain important clinical considerations for long-term outcome.

## Figures and Tables

**Figure 1:**
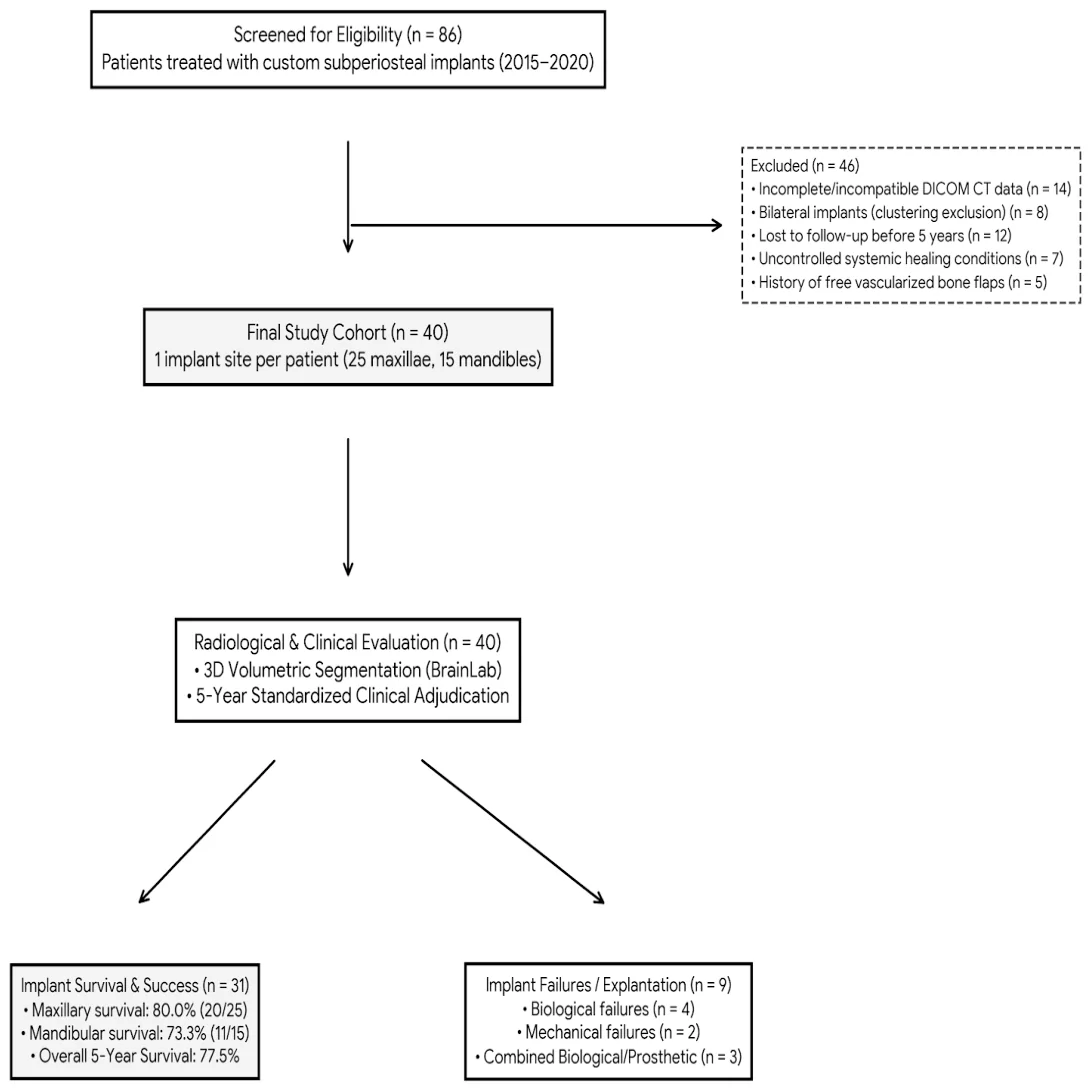
STROBE (Strengthening the Reporting of Observational Studies in Epidemiology) participant flow diagram.

**Figure 2:**
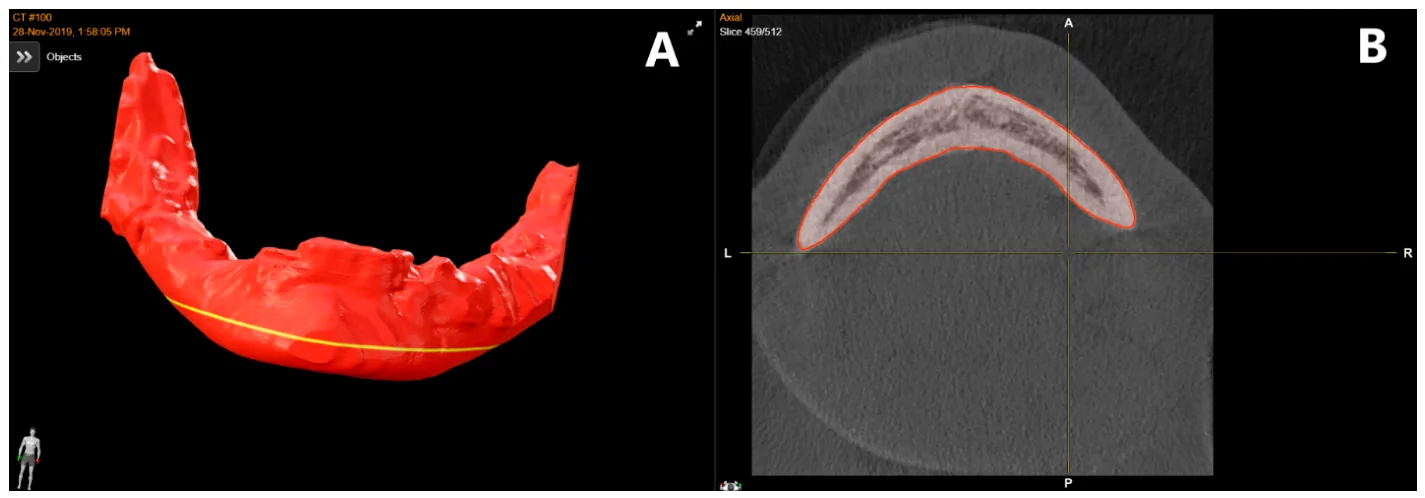
Representative 3D and 2D radiological segmentation of an edentulous, severely atrophic mandible. Panel (**A**) displays a 3D volumetric reconstruction of the residual mandibular bone (segmented in red using BrainLab software) evaluated for subperiosteal implant planning. Panel (**B**) shows a corresponding axial CBCT slice with a red outline defining the cross-sectional skeletal boundary. The standardized segmentation protocols available in the BrainLab platform enable consistent volume quantification and support measurement reproducibility between independent assessors.

**Figure 3:**
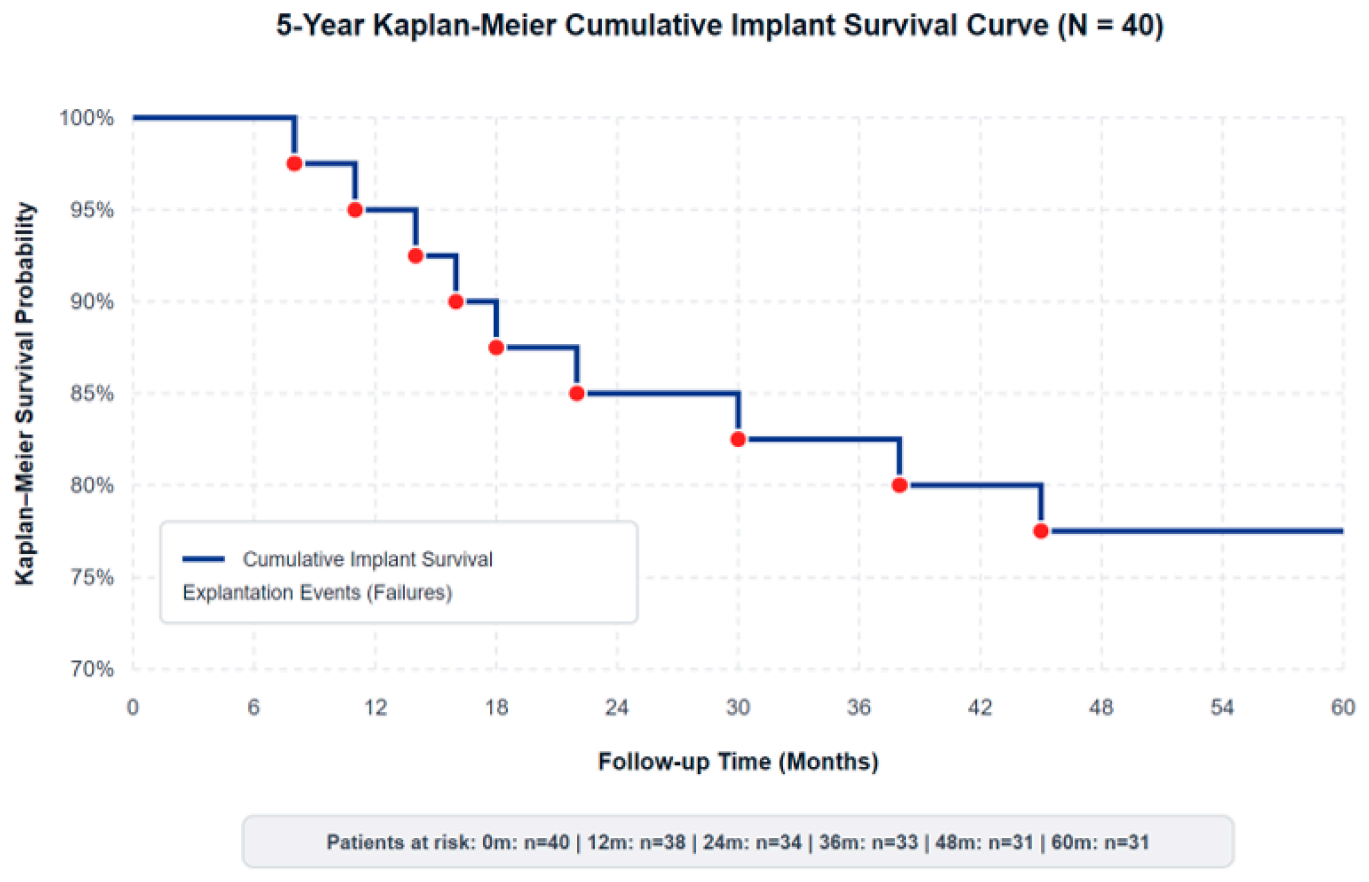
Five-year Kaplan–Meier cumulative subperiosteal implant survival curve for the complete-case cohort (N = 40). Red markers represent total framework explantation events. Numbers at risk at specified time points: 0 months: *n* = 40; 12 months: *n* = 38; 24 months: *n* = 34; 36 months: *n* = 33; 48 months: *n* = 31; 60 months: *n* = 31.

**Figure 4:**
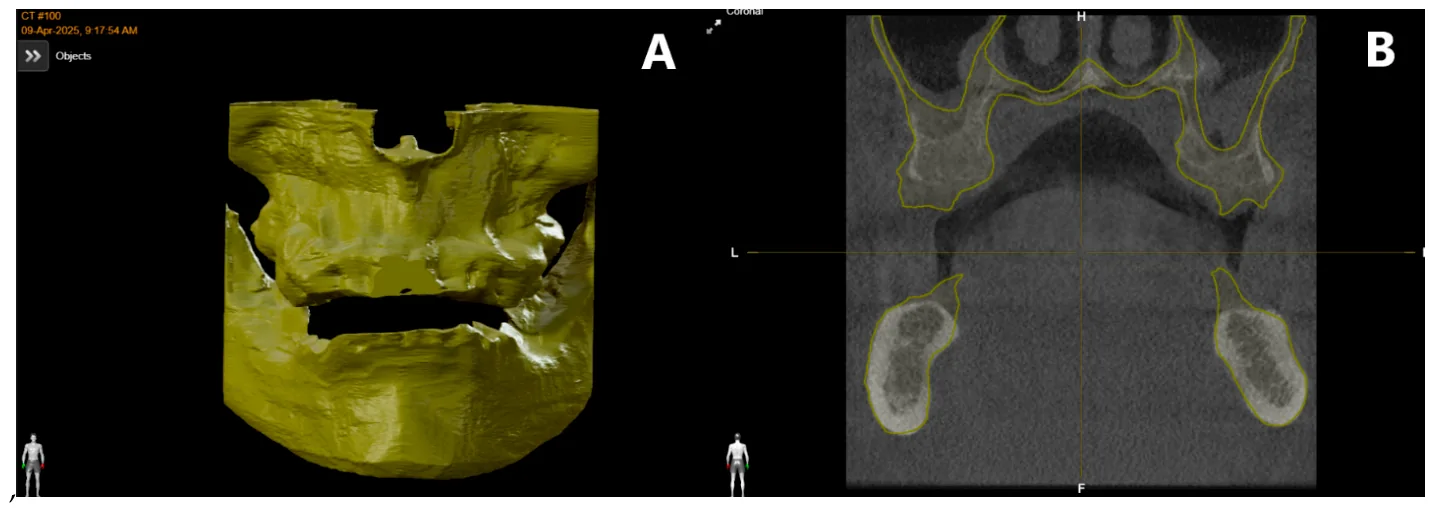
Multi-planar visualization and baseline skeletal segmentation for 3D bone volume quantification. Panel (**A**) shows the initial 3D digital reconstruction of an atrophic maxillofacial complex. Panel (**B**) demonstrates the corresponding coronal CBCT cross-section with yellow contour lines delineating the anatomical boundaries of the maxilla and mandible, excluding surrounding soft tissues and non-skeletal structures.

**Table 1:** Clinical data.

Variables		Survival (*n* = 31)		Failure (*n* = 9)		*p*-Value
Sex (F; M)		F-10	M-21	F-4	M-5	*p* = 0.694 *
Age (years)		64 (IQR 57–68)		60 (IQR 58–66)		*p* = 0.588 **
Procedure duration (minutes)		92 (IQR 76–96)		87 (IQR 67–106)		*p* = 0.483 **
Preoperative Cawood and Howell scale	IV	8		0		*p* = 0.327 *
	V	13		5		
	VI	10		4		
Oral hygiene (0—negative; 1—positive)	0	2		4		***p*** = 0.007 *
	1	29		5		
Medical comorbidities (yes; no)		Y-8	N-23	Y-1	N-8	*p* = 0.654 *
Smoking (yes; no)		Y-5	N-26	Y-6	N-3	***p*** = 0.007 *
Medications (yes; no)		Y-11	N-20	Y-2	N-7	*p* =0.690

* Fisher’s exact test. ** Mann–Whitney U test.

**Table 2:** Individual characteristics of the nine explanted frameworks.

Patient ID.	Location	Time to Failure (Months)	Primary Failure Category	Secondary/Contributing Factors	Clinical Presentation & Surgical Findings	Management/Outcome
#2	Mandible	8	Biological	Heavy smoking (>20 cig/day), poor hygiene	Severe purulent infection, extensive mucosal necrosis, strut exposure	Refractory to debridement; Total Explantation
#7	Maxilla	11	Biological/Mechanical	Severe occlusal overload, lack of keratinized tissue	Strut exposure in anterior zone, rapid bone loss around screws	Partial resection failed; Total Explantation
#12	Mandible	14	Biological	Heavy smoking, severe peri-implantitis	Widespread soft-tissue dehiscence, chronic suppuration	Anti-inflammatory therapy failed; Total Explantation
#19	Maxilla	16	Biological	Oro-antral communication, sinus involvement	Major mucosal breakdown in premolar area, maxillary sinusitis	Flap coverage failed; Total Explantation
#22	Maxilla	18	Combined (Biological/Prosthetic)	Peri-implantitis, bone resorption around screws	Mobile fixation screws, progressive osteolysis surrounding 4/6 screws	Screw removal failed; Total Explantation
#28	Mandible	22	Mechanical	Fixation screw loosening in atrophic bone	Secondary framework mobility, persistent pain on mastication	Re-fixation impossible; Total Explantation
#31	Maxilla	30	Mechanical/Prosthetic	Prosthetic abutment fracture, screw shear failure	Broken anterior prosthetic post, framework instability	Structural repair impossible; Total Explantation
#34	Maxilla	38	Biological	Heavy smoking, chronic mucosal breakdown	Extensive soft-tissue necrosis, large bare metal exposure	Flap plastic revision failed; Total Explantation
#37	Mandible	45	Mechanical	Structural strut fracture in canine region	Fracture of main body strut, secondary chronic infection	Unstable framework; Total Explantation

**Table 3:** Subgroup comparison of preoperative residual bone volume (cm^3^) between surviving and failed custom subperiosteal implants stratified by Cawood & Howell anatomical classes.

Anatomical Site & Cawood Class	Survival Group Volume, cm^3^, Median (IQR) [n/N]	Failure Group Volume, cm^3^, Median (IQR) [n/N]	Statistical Test Applied	Effect Size Estimate (r)	*p*-Value
Maxilla Volumetric Assessment (N = 40)					
Class IV	14.2 (13.9–14.2) [*n* = 8/8]	No failures [*n* = 0/8]	N/A (No failure cases)	N/A	N/A
Class V	12.7 (12.4–13.1) [*n* = 13/18]	11.8 (11.5–12.2) [*n* = 5/18]	Mann–Whitney U test	r=0.88 (Large)	0.003
Class VI	12.0 (11.5–12.2) [*n* = 10/14]	10.9 (10.6–10.9) [*n* = 4/14]	Mann–Whitney U test	r=1.00 (Large)	0.002
Mandible Volumetric Assessment (N = 40)					
Class IV	24.4 (24.2–24.9) [*n* = 8/8]	No failures [*n* = 0/8]	N/A (No failure cases)	N/A	N/A
Class V	23.7 (23.5–24.0) [*n* = 13/18]	21.9 (21.2–22.4) [*n* = 5/18]	Mann–Whitney U test	r=1.00 (Large)	<0.001
Class VI	22.5 (21.1–23.2) [*n* = 10/14]	20.7 (20.4–21.2) [*n* = 4/14]	Mann–Whitney U test	r=0.68 (Moderate)	0.054

All subgroup comparisons represent exploratory secondary analyses. N/A = Not applicable (no failure events observed in Class IV). Effect size r calculated as $r=\frac{\left|Z\right|}{\surd N}$. Continuous volume data presented as median (IQR).

## Data Availability

The datasets generated and analyzed during the current study are available from the corresponding author upon reasonable academic request, subject to patient privacy constraints.
